# Supplementary material for: Prolyl hydroxylase substrate adenylosuccinate lyase is an oncogenic driver in triple negative breast cancer
Source: Nat Commun. 2019 Nov 15;10:5177. doi: 10.1038/s41467-019-13168-4 (PMC6858455; doi:10.1038/s41467-019-13168-4)
Supplement: Supplementary file 1 — Supplementary Information [file 41467_2019_13168_MOESM1_ESM.pdf]

Prolyl Hydroxylase Substrate Adenylosuccinate Lyase Is An Oncogenic Driver In Triple Negative Breast Cancer

Zurlo et al.

Supplementary Information

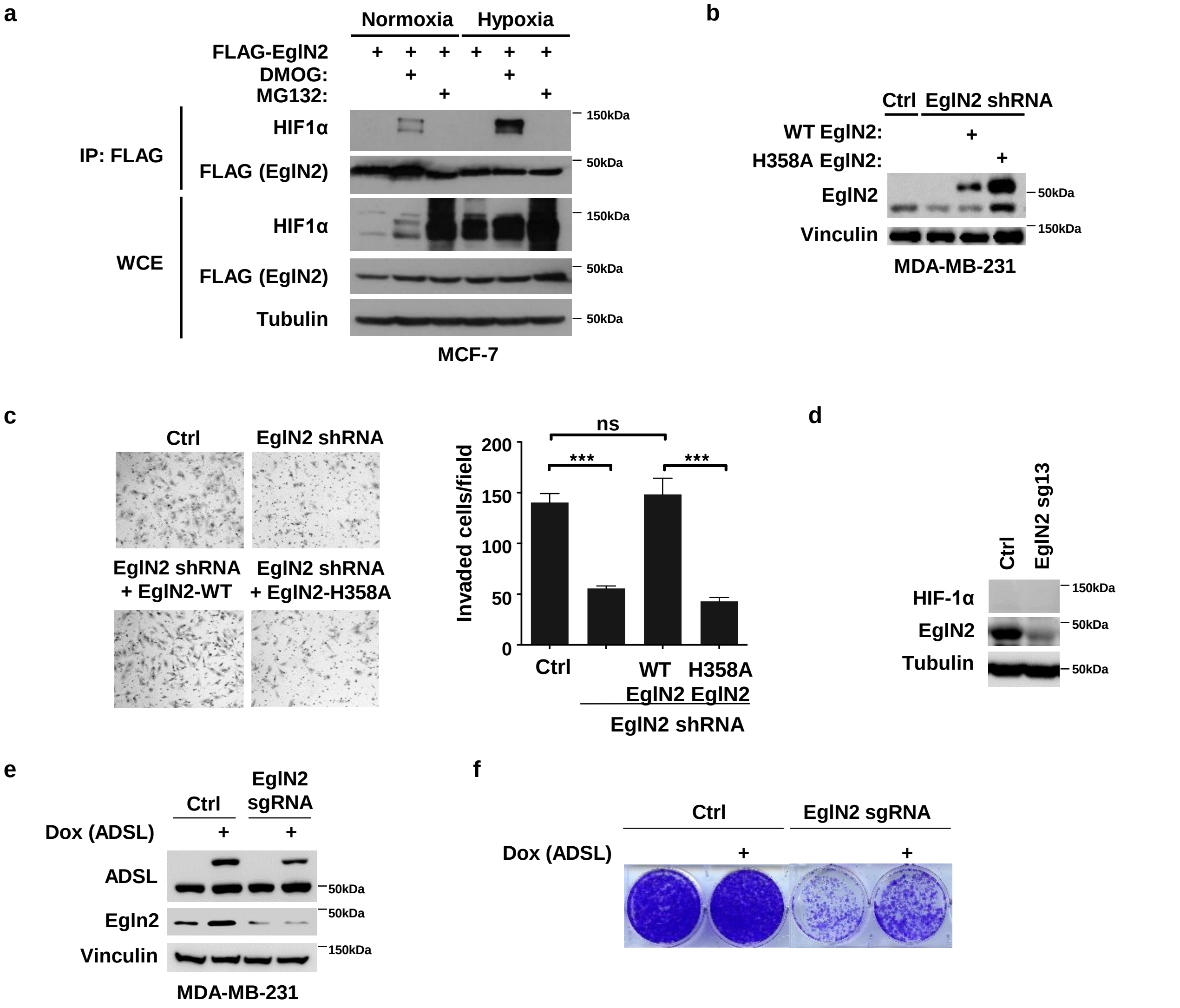

Supplementary Figure 1. Substrate-trapping strategy identifies ADSL as an EglN2 substrate in TNBC. (a) Immunoblots (IB) of whole-cell extracts (WCE) and immunoprecipitations (IP) of lysates from MCF-7 cells infected with lentivirus encoding FLAG-tagged EglN2, and treated as indicated overnight. Hypoxia was 1% O_2, DMOG concentration was 1mM, and MG132 was used at 10µM. **(b)** IB of WCE of lysates from control- or EglN2 shRNA-transduced MDA-MB-231 cells, subsequently infected with lentivirus encoding either EglN2-WT or -H358A. **(c)** Representative images of invasion of MDA-MB-231 cells described in (b). Graphs represent the mean ± SEM from three independent experiments. **(d)** IB of WCE of lysates from MDA-MB-231 cells transduced with indicated lentivirus. **(e)** IB of WCE of lysates from MDA-MB-231 cells transduced with indicated lentivirus and treated as indicated for 5 days. **(f)** Representative 2-D proliferation of MDA-MB-231 cells described in (e). In (e) and (f), dox concentration was 2µM. Source data are provided as a Source Data file.

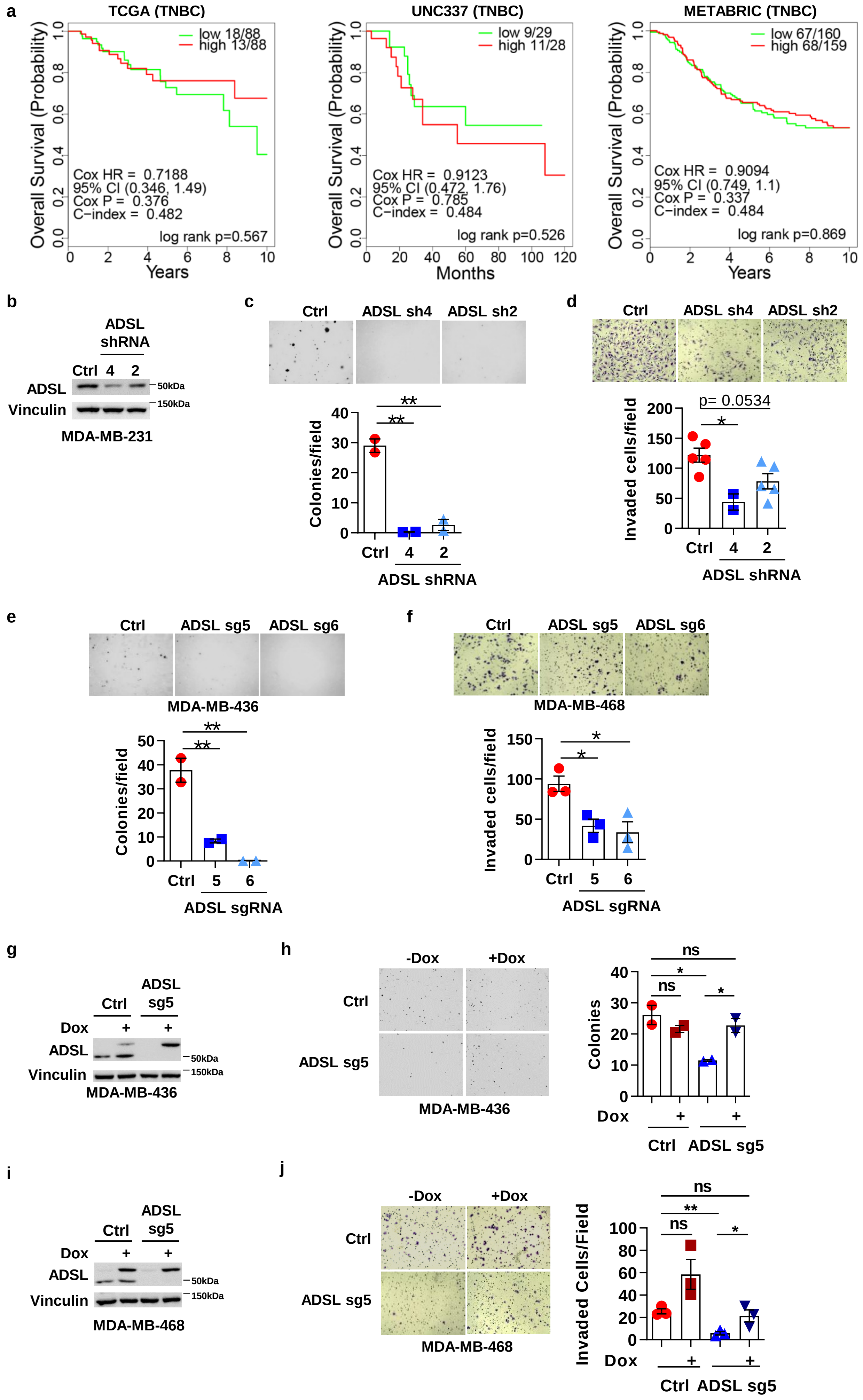
a
TCGA (TNBC)
UNC337 (TNBC)
METABRIC (TNBC)
Overall Survival (Probability)
low 18/88
high 13/88
Cox HR = 0.7188
95% CI (0.346, 1.49)
Cox P = 0.376
C−index = 0.482
log rank p=0.567
Years
low 9/29
high 11/28
Cox HR = 0.9123
95% CI (0.472, 1.76)
Cox P = 0.785
C−index = 0.484
log rank p=0.526
Months
low 67/160
high 68/159
Cox HR = 0.9094
95% CI (0.749, 1.1)
Cox P = 0.337
C−index = 0.484
log rank p=0.869
Years
b
ADSL shRNA
Ctrl 4 2
ADSL
Vinculin
50kDa
150kDa
MDA-MB-231
c
Ctrl
ADSL sh4
ADSL sh2
Colonies/field
ADSL shRNA
d
Invaded cells/field
p= 0.0534
ADSL shRNA
e
ADSL sg5
ADSL sg6
MDA-MB-436
Colonies/field
ADSL sgRNA
f
MDA-MB-468
Invaded cells/field
ADSL sgRNA
g
Ctrl
ADSL sg5
Dox
ADSL
Vinculin
MDA-MB-436
h
-Dox
+Dox
Colonies
ns
i
MDA-MB-468
j
Invaded Cells/Field

Supplementary Figure 2. ADSL plays an essential role in TNBC. (a) Correlation between ADSL mRNA expression and overall TNBC patient survival probability across different subtypes of breast cancer in three different datasets (TCGA stands for The Cancer Genome Atlas). The green line represents low ADSL expression, whereas the red line represents high ADSL expression. **(b)** IB of lysates from MDA-MB-231 cells transduced with indicated lentivirus. **(c and d)** Representative images of (c) anchorage-independent growth and (d) invasion of MDA-MB-231 cells upon ADSL depletion shown by IB in (b). Graphs represent the mean ± SEM from two independent experiments, each performed in duplicate (c), and from at least two independent experiments (d). Red circles represent control (ctrl) cells, blue squares ADSL shRNA #4 and light blue triangles ADSL shRNA #2 cells. **(e and f)** Representative images of (e) anchorage-independent growth of MDA-MB-436 cells and (f) invasion of MDA-MB-468 cells upon ADSL depletion by two independent sgRNAs (#5 and #6). Graphs represent the mean ± SEM from two independent experiments, each performed in duplicate (e), and from three independent experiments (f). Red circles represent control (ctrl) cells, blue squares ADSL sgRNA #5 and light blue triangles ADSL sgRNA #6 cells. **(g)** IB of lysates from MDA-MB-436 cells overexpressing doxycycline (dox)-inducible ADSL, infected with indicated virus and treated as indicated. **(h)** Representative images of anchorage-independent growth of MDA-MB-436 cells described in (g). Graphs represent the mean ± SEM from two independent experiments, each performed in duplicate **(i)** IB of lysates from MDA-MB-468 cells overexpressing doxycycline (dox)-inducible ADSL, infected with indicated virus and treated as indicated. **(j)** Representative images of invasion of MDA-MB-468 cells described in (i). Graphs represent the mean ± SEM from three independent experiments. In (h) and (j), red circles represent control cells, dark red squares control treated with dox, blue triangles ADSL sgRNA #5 cells, dark blue triangles ADSL sgRNA #5 treated with dox. In (c), (d), (e) and (f), $^{*}P < 0.05$, $^{**}P < 0.01$, $^{***}P < 0.001$ were calculated using one-way ANOVA followed by Dunnett’s multiple comparison test. Source data are provided as a Source Data file.

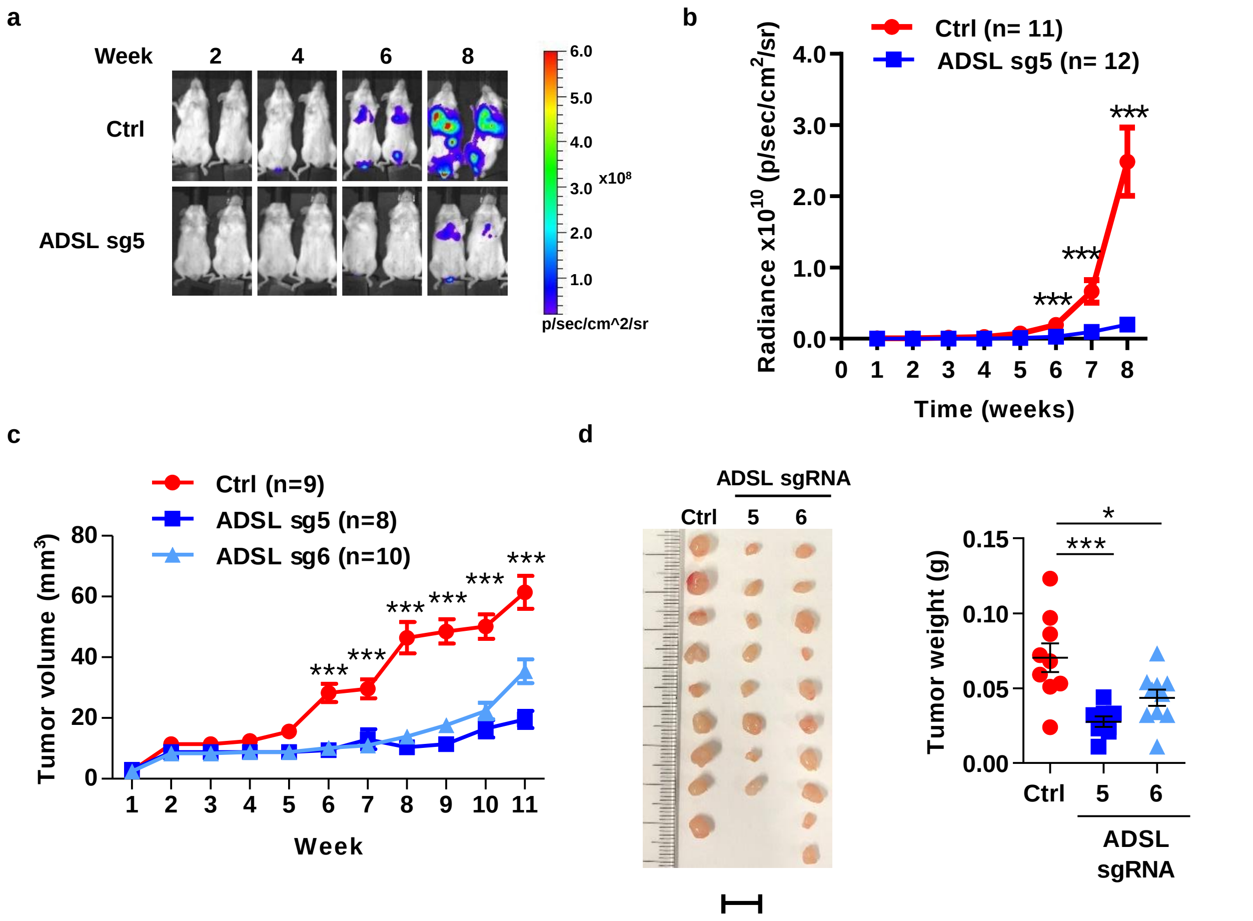

Supplementary Figure 3. ADSL is required for TNBC tumorigenesis and lung colonization. (a) Representative bioluminescence images from the indicated weeks after the orthotopical injection of MDA-MB-231 luciferase-expressing cells transduced with lentivirus expressing either ADSL sgRNA #5 (sg5) or control sgRNA (Ctrl) into the mammary fat pads of NOD SCID Gamma (NSG) mice. **(b)** Quantification of the bioluminescence imaging. **(c)** Quantification of the tumor volume from the indicated weeks after the orthotopical injection of MDA-MB-468 cells transduced with lentivirus expressing either ADSL sgRNA #5 (sg5), ADSL sgRNA #6 (sg6) or control sgRNA (Ctrl) into the mammary fat pads of NOD SCID Gamma (NSG) mice. **(d)** Image of tumors after dissection and quantification of tumor weight. Scale bar represents 1cm. In (b), (c) and (d), the red circles represent control (ctrl) cells, blue squares ADSL sg5, and light blue triangles ADSL sg6. For (b) and (c), the Mann-Whitney test was used to calculate the P values. For (d), P values were calculated using one-way ANOVA followed by Dunnett's multiple comparison test. Error bars represent SEM, *$P < 0.05$, **$P < 0.01$, ***$P < 0.001$. Source data are provided as a Source Data file.

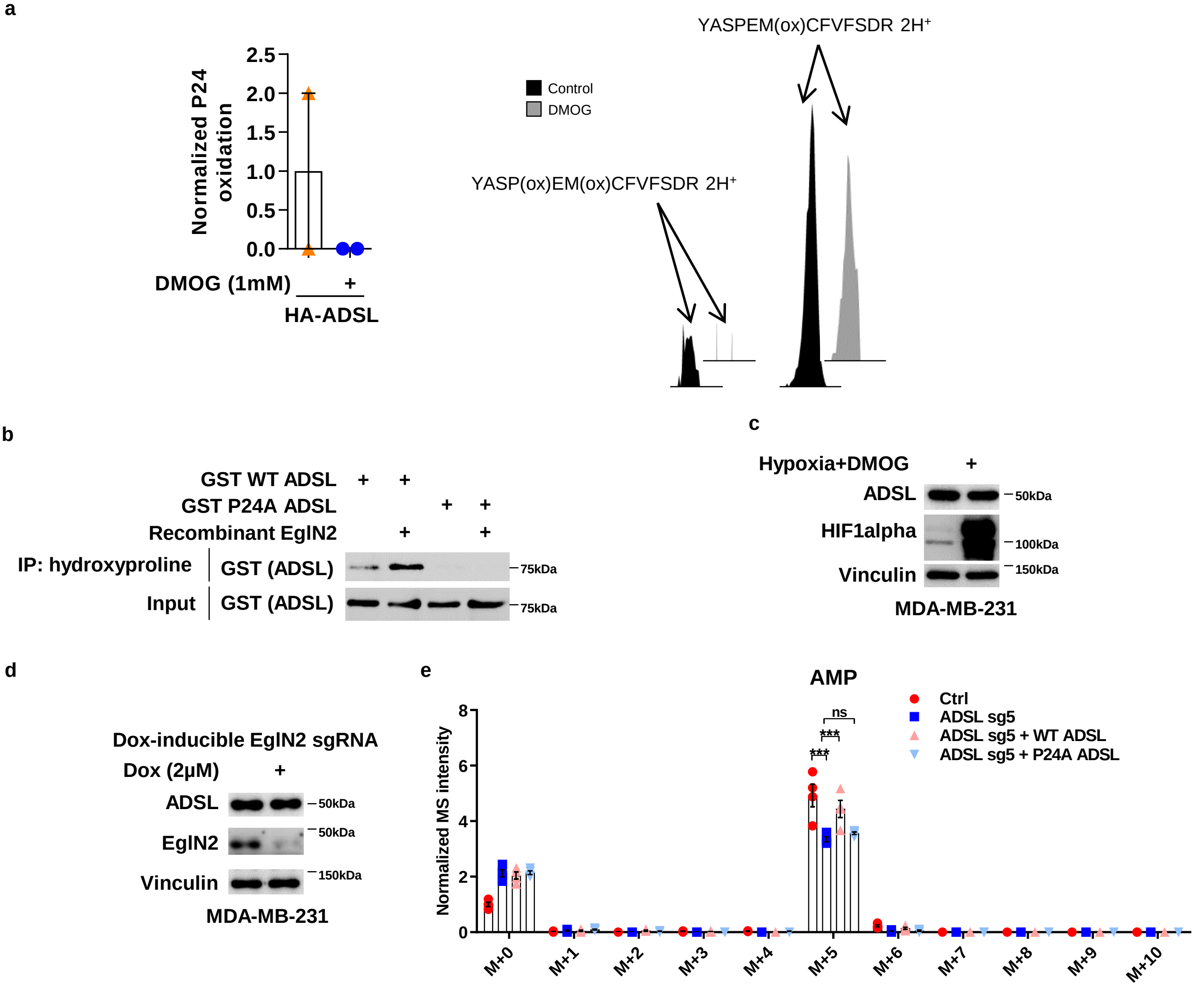

Supplementary Figure 4. ADSL is hydroxylated by EglN2 on Proline 24. (a) ADSL proline 24 hydroxylation in the presence or absence of the hydroxylase inhibitor DMOG. Bar graph represents the normalized ratio of the intensity of the oxidized P24-containing peptide to that of ADSL full protein. Error bars represent SEM, n= 2. **(b)** IB of input and IP of in vitro hydroxylation reactions of eluted GST WT and P24A ADSL in the presence or absence of recombinant EglN2. **(c)** IB of lysates from MDA-MB-231, treated as indicated overnight. Hypoxia was 1%O_2, and DMOG was used at 1mM. **(d)** IB of lysates from MDA-MB-231, treated as indicated for at least 48h. **(e)** Normalized mass spectrometry (MS) intensity of ^{13}C-labeled AMP isotopes in cells transduced with the indicated lentiviruses and supplemented with [U-$^{13}C_6$]glucose for 24h. Error bars represent SEM from four independent sets of samples, ***P<0.001 were calculated using two-way ANOVA followed by Tukey’s multiple comparison test. Source data are provided as a Source Data file.

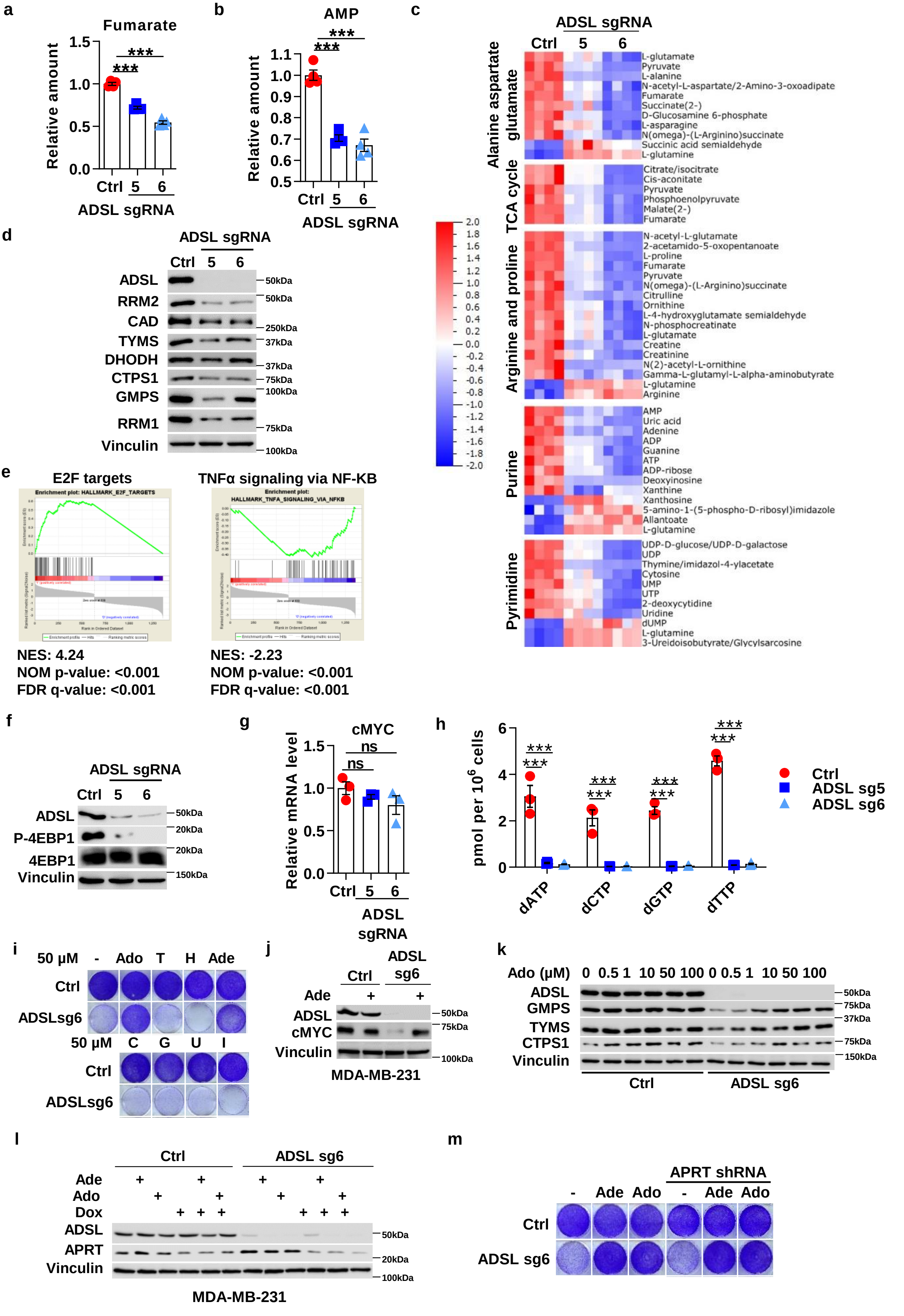

a
Fumarate
Relative amount
Ctrl 5 6
ADSL sgRNA
b
AMP
Relative amount
Ctrl 5 6
ADSL sgRNA
c
ADSL sgRNA
Ctrl 5 6
Alanine aspartate glutamate
L-glutamate
Pyruvate
L-alanine
N-acetyl-L-aspartate/2-Amino-3-oxoadipate
Fumarate
Succinate(2-)
D-Glucosamine 6-phosphate
L-asparagine
N(omega)-(L-Arginino)succinate
Succinic acid semialdehyde
L-glutamine
TCA cycle
Citrate/isocitrate
Cis-aconitate
Pyruvate
Phosphoenolpyruvate
Malate(2-)
Fumarate
Arginine and proline
N-acetyl-L-glutamate
2-acetamido-5-oxopentanoate
L-proline
Fumarate
Pyruvate
N(omega)-(L-Arginino)succinate
Citrulline
Ornithine
L-4-hydroxyglutamate semialdehyde
N-phosphocreatinate
L-glutamate
Creatine
Creatinine
N(2)-acetyl-L-ornithine
Gamma-L-glutamyl-L-alpha-aminobutyrate
L-glutamine
Arginine
Purine
AMP
Uric acid
Adenine
ADP
Guanine
ATP
ADP-ribose
Deoxyinosine
Xanthine
Xanthosine
5-amino-1-(5-phospho-D-ribosyl)imidazole
Allantoate
L-glutamine
Pyrimidine
UDP-D-glucose/UDP-D-galactose
UDP
Thymine/imidazol-4-ylacetate
Cytosine
UMP
UTP
2-deoxycytidine
Uridine
dUMP
L-glutamine
3-Ureidoisobutyrate/Glycylsarcosine
d
ADSL sgRNA
Ctrl 5 6
ADSL
RRM2
CAD
TYMS
DHODH
CTPS1
GMPS
RRM1
Vinculin
e
E2F targets
TNFα signaling via NF-KB
NES: 4.24
NOM p-value: <0.001
FDR q-value: <0.001
NES: -2.23
NOM p-value: <0.001
FDR q-value: <0.001
f
ADSL sgRNA
Ctrl 5 6
ADSL
P-4EBP1
4EBP1
Vinculin
g
cMYC
Relative mRNA level
Ctrl 5 6
ADSL sgRNA
h
pmol per 10^6 cells
dATP
dCTP
dGTP
dTTP
Ctrl
ADSL sg5
ADSL sg6
i
50 μM - Ado T H Ade
Ctrl
ADSLsg6
50 μM C G U I
j
Ctrl
ADSL sg6
Ade
ADSL
cMYC
Vinculin
MDA-MB-231
k
Ado (μM) 0 0.5 1 10 50 100 0 0.5 1 10 50 100
ADSL
GMPS
TYMS
CTPS1
Vinculin
Ctrl
ADSL sg6
l
Ctrl
ADSL sg6
Ade
Ado
Dox
ADSL
APRT
Vinculin
MDA-MB-231
m
APRT shRNA
- Ade Ado - Ade Ado
Ctrl
ADSL sg6

Figure 5. ADSL controls cMYC protein level by regulating adenosine levels. (a and b) Relative amount of the metabolite (a) fumarate and (b) AMP in control or ADSL-depleted MDA-MB-231 cells. Graphs represent the mean ± SEM from four independent sets of samples. ***P < 0.001 were calculated using one-way ANOVA followed by Dunnett's multiple comparison test. **(c)** Heat map representing the metabolomics analysis in control or ADSL-depleted MDA-MB-231 cells in the indicated pathways. **(d)** IB of lysates from MDA-MB-231 cells, transduced with indicated lentivirus. **(e)** Gene Set Enrichment Analysis (GSEA) of E2F target genes and TNFα signaling via NF-kB between control and ADSL knockout MDA-MB-231 cells. **(f)** IB of lysates from MDA-MB-231 cells, transduced with indicated lentivirus. **(g)** Relative cMYC mRNA level lysates in control or ADSL-depleted MDA-MB-231 cells. Bar graphs represent the mean ± SEM from three independent sets of samples. P value (ns= non significant) were calculated using one-way ANOVA followed by Dunnett's multiple comparison test. **(h)** dNTP levels in control or ADSL-depleted MDA-MB-231 cells. Bar graphs represent the mean ± SEM from three independent sets of samples. ***P < 0.001 were calculated using two-way ANOVA followed by Dunnett's multiple comparison test. **(i)** Representative images of 2-d proliferation of MDA-MB-231 cells, transduced and treated as indicated for at least 5 days. Ado= adenosine, T= thymidine, H= hypoxanthine, Ade= adenine, C= cytidine, G=guanosine, U= uridine, I= inosine. **(j, k and l)** IB of lysates from MDA-MB-231 cells, transduced with indicated lentivirus and treated as indicated. In (j) and (l) adenine was used at 50μM. In (l) adenosine concentration was 50μM, and dox was used at 2μM. **(m)** Representative images of 2-d proliferation of MDA-MB-231 cells, transduced and treated as indicated for at least 5 days. Adenosine and adenine were used at 50μM. Source data are provided as a Source Data file.

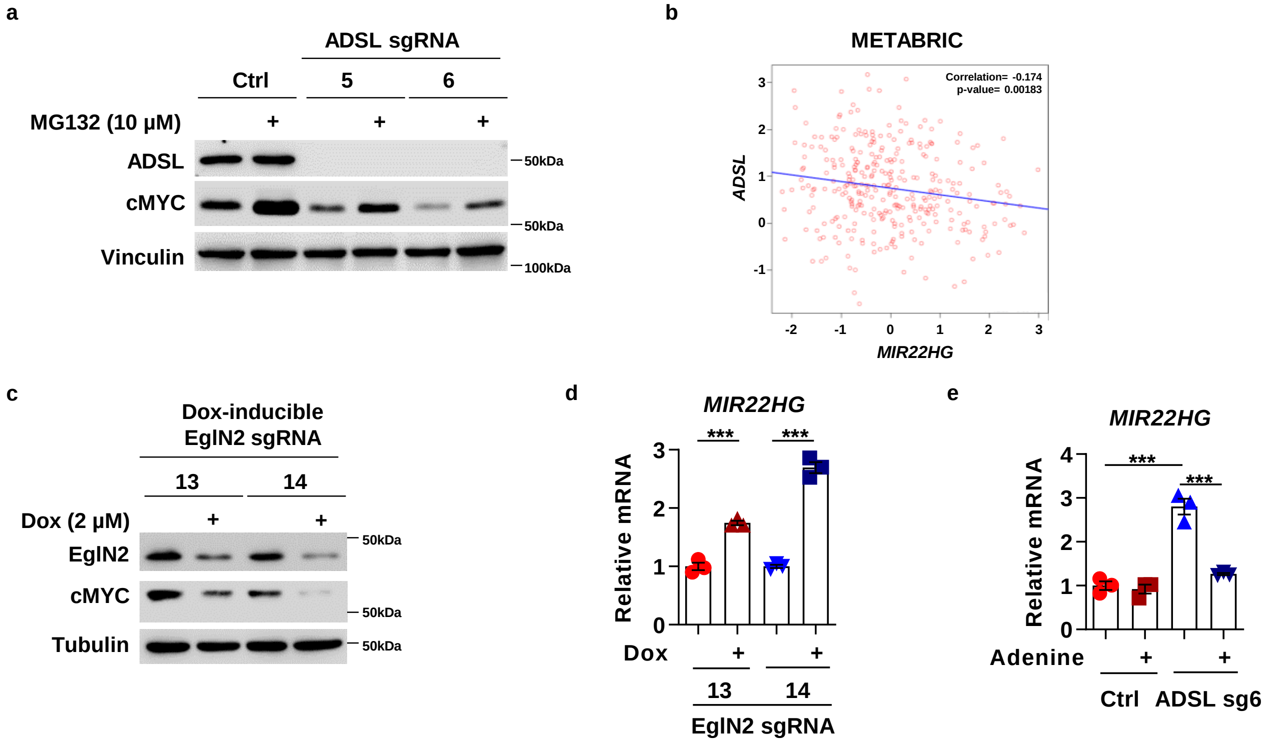

Supplementary Figure 6. ADSL controls cMYC negative regulator *MIR22HG* expression. (a) IB of lysates from MDA-MB-231 cells, transduced with indicated lentivirus and treated as indicated overnight. **(b)** Pearson correlation between the expression of *ADSL* and *MIR22HG* in TNBC patients from METABRIC dataset. **(c)** IB of lysates from MDA-MB-231 cells, transduced with indicated lentivirus and treated as indicated overnight. **(d and e)** *MIR22HG* mRNA expression in (d) dox-inducible EglN2 sgRNA MDA-MB-231 cells in the presence or absence of dox (2µM) and (f) ADSL control or knockout MDA-MB-231 cells treated as indicated (adenine concentration was 50µM). Graphs represent the mean ± SEM from three independent sets of samples. **P < 0.01, ***P < 0.001 were calculated using one-way ANOVA followed by Tukey's multiple comparison test. Source data are provided as a Source Data file.

Supplementary Table 1: List of potential EglN2 substrates from TAP-TAG purification

		WT EglN2			
		N		H+D	
**Accession**	**Description**	**# PSMs**	**# PSMs**	**# PSMs**	**# PSMs**
**Q96KS0**	**Egl nine homolog 2 (EglN2)**	**27**	**29**	**25**	**25**
P30566	Adenylosuccinate lyase	1	1	7	5
P11021	78 kDa glucose-regulated protein	0	0	2	2
H0Y5H9	Serpin B4	1	0	3	3
P31947-2	14-3-3 protein sigma	0	0	3	3
O14744	Protein arginine N-methyltransferase 5	5	4	8	8
P15924	Desmoplakin	10	10	17	18
Q5HY54	Filamin-A	3	2	8	8
P16403	Histone H1.2	0	0	2	3
P81605	Dermcidin	1	1	2	2
A0A075B6L0	Ig lambda-3 chain C region	1	1	2	2

N= normoxia, H+D= hypoxia (1%O_2) + DMOG (1mM), MDA-MB-231 treated overnight. PSM= Peptide Spectrum Match.

Supplementary Table 2: List of potential EglN2 substrates from GST pull-down

		Ctrl		WT EglN2	
		N	H+D	N	H+D
**Accession**	**Description**	**# PSMs**	**# PSMs**	**# PSMs**	**# PSMs**
Q96KS0	**Egl nine homolog 2 (EglN2)**	**0**	**0**	**327**	**288**
P30566	Adenylosuccinate lyase	0	0	1	16
P22061	Protein-L-isoaspartate(D-aspartate) O-methyltransferase	0	0	11	12
O00622	Protein CYR61	0	0	0	1
O00505	Importin subunit alpha-4	0	0	4	7
Q9Y3D0	Mitotic spindle-associated MMXD complex subunit MIP18	0	0	1	4
P52294	Importin subunit alpha-5	0	0	3	4
O15371	Eukaryotic translation initiation factor 3 subunit D	0	0	0	1
Q9BQ61	Uncharacterized protein C19orf43	0	0	0	1
P56537	Eukaryotic translation initiation factor 6	0	0	1	2
P46779	60S ribosomal protein L28	0	0	0	1
O14556	Glyceraldehyde-3-phosphate dehydrogenase, testis-specific	0	0	1	2
Q15369	Transcription elongation factor B polypeptide 1	0	0	1	3

N= normoxia, H+D= hypoxia (1%O_2) + DMOG (1mM), MDA-MB-231 treated overnight. PSM= Peptide Spectrum Match.

Supplementary Table 3: Pearson correlation between *ADSL* and *cMYC* target expression

	METABRIC (TNBC)		TCGA (TNBC)	
cMYC target	Correlation vs ADSL	p-value	Correlation vs ADSL	p-value
BUB3	0.146	0.00912	0.224	0.00269
CAD	0.231	2.90E-05	0.122	0.16
CBX3	-0.0117	0.835	0.212	0.00459
CCT2	0.28	3.59E-07	0.258	0.000538
CCT5	0.00598	0.915	0.257	0.000541
CDC20	0.32	5.12E-09	0.397	4.42E-08
CDC45	0.428	1.19E-15	0.523	8.33E-14
EIF4H	0.0625	0.266	0.148	0.0495
GNL3	0.161	0.00401	0.244	0.00105
HDAC2	0.158	0.0047	0.234	0.00174
HNRNPA1	0.301	4.37E-08	0.294	7.17E-05
HNRNPA2B1	0.174	0.00178	0.159	0.0344
HNRNPA3	0.235	2.32E-05	0.197	0.00849
HNRNPC	0.1	0.0743	0.208	0.00555
HNRNPD	0.203	0.000262	0.284	0.000127
HPRT1	0.381	1.96E-12	0.314	2.08E-05
HSP90AB1	0.142	0.0111	0.151	0.0441
HSPD1	0.282	3.12E-07	0.293	7.72E-05
KPNA2	0.102	0.0688	0.341	3.37E-06
LDHA	0.131	0.0191	0.185	0.0138
MCM2	0.268	1.18E-06	0.266	0.000352
MCM6	0.339	4.99E-10	0.211	0.0048
NAP1L1	0.161	0.00387	0.279	0.000167
NPM1	0.163	0.00344	0.302	4.43E-05
ODC1	0.32	5.07E-09	0.368	4.74E-07
PCNA	0.23	3.44E-05	0.304	3.93E-05
PGK1	0.0244	0.6641	-0.0145	0.848
PSMD1	0.158	0.0048	0.132	0.0793
PSMD14	0.0925	0.0991	0.234	0.00175
RRM1	0.211	0.000148	0.11	0.146
SERBP1	0.239	1.63E-05	0.367	5.20E-07
SET	0.162	0.00375	0.213	0.00438
SF3B3	0.122	0.0297	0.206	0.006
SRSF1	0.199	0.000354	0.251	0.00077
SRSF2	0.262	2.09E-06	0.362	7.45E-07
SRSF3	0.2	0.000317	0.413	1.11E-08
SRSF7	0.061	0.277	0.275	0.000206
TARDBP	-0.0177	0.753	0.182	0.0151
TYMS	0.116	0.038	0.283	0.000134
TCP1	0.385	9.51E-13	0.347	2.28E-06
VDAC1	0.0508	0.365	0.0558	0.46

Supplementary Table 4: List of the genes mostly affected by ADSL depletion via sgRNA5

Gene	Ctrl	Ctrl	Ctrl	ADSL sg5	ADSL sg5	ADSL sg5	log2 fold change	Adjusted p-value
**MIR22HG**	**8.949732752**	**8.994610217**	**8.942760017**	**10.3655576**	**10.42428466**	**10.41816602**	**1.480849597**	**1.45E-67**
HERPUD1	10.53226527	10.46203103	10.54989361	11.63906468	11.65230738	11.64814542	1.143171841	4.84209E-47
HNRNPA1	13.79473542	13.80621439	13.74888778	12.81330421	12.87135803	12.81023506	-0.953598248	3.00788E-46
LBH	7.362889819	7.562071609	7.60764557	9.055690139	9.054595006	9.025981131	1.64571505	1.58974E-42
SRXN1	11.03975013	11.07348865	11.06171855	11.806495	11.80319808	11.76828533	0.740214147	9.05868E-38
CDKN3	10.41202259	10.33019265	10.3209459	9.033633986	9.144412903	9.061755733	-1.308123706	3.59774E-35
LOC105370526	6.12223629	5.853797218	6.117637921	7.870936651	7.862829927	7.956869162	2.253156568	5.31063E-35
NPM1	15.55736951	15.60045621	15.52688603	14.74513599	14.8269575	14.8121489	-0.767144386	1.68963E-33
HNRNPA1P10	11.87305731	11.88294094	11.82257496	10.980302	10.92533092	10.85156293	-0.947537175	3.01837E-33
HSPB8	9.501454145	9.373018321	9.4728103	10.5912665	10.58828644	10.61536811	1.173581025	3.20929E-33
GABARAPL1	9.463563661	9.44970328	9.344496569	10.40769535	10.41245543	10.40442706	1.011430145	4.39506E-32
AURKA	13.18376779	13.12929622	13.15632806	12.44901517	12.51357128	12.49833006	-0.671312916	6.55088E-32
SRSF7	11.68305527	11.71903172	11.62929902	10.63778256	10.69235517	10.66815724	-1.021019461	9.81759E-32
RHOB	8.734531971	8.821446441	9.038639551	10.46595257	10.28643886	10.3036717	1.526733911	5.74193E-31
CSF1	10.96789069	10.9614975	10.86919461	11.75955386	11.69099494	11.69806011	0.789787183	2.69822E-29
CDCA8	11.70951919	11.66284883	11.6704069	10.94494585	10.93908447	10.98205613	-0.731871582	3.46511E-29
SDC3	11.51161794	11.52535669	11.44705712	10.65256734	10.65938033	10.57439877	-0.874634933	5.74718E-29
NEDD9	9.211259889	9.155269325	9.183578886	10.02897275	10.07171601	10.06165432	0.896202972	3.79315E-28
SLC6A12	4.825669386	4.855555401	4.625226555	7.123087236	7.225614095	7.295680624	3.695668793	1.11943E-27
TSC22D3	8.155076548	8.15582559	8.087874992	9.501929644	9.513873049	9.545806952	1.458798728	3.6129E-27

Supplementary Table 5: List of the genes most affected by ADSL depletion via sgRNA6

Gene	Ctrl	Ctrl	Ctrl	ADSL sg6	ADSL sg6	ADSL sg6	log2 fold change	Adjusted p-value
**MIR22HG**	**8.94973275**	**8.99461022**	**8.94276002**	**10.9095004**	**10.8377282**	**10.9500019**	**1.983876125**	**1.362E-123**
HERPUD1	10.5322653	10.462031	10.5498936	11.7963755	11.891466	11.9472782	1.378310836	4.2101E-69
AURKA	13.1837678	13.1292962	13.1563281	12.1761486	12.1747351	12.2119131	-0.972175128	2.3697E-66
LBH	7.36288982	7.56207161	7.60764557	9.39168658	9.37032365	9.39606817	1.998715357	1.5137E-63
CYP1A1	9.39454309	9.51540204	9.59890361	11.1795845	11.3708152	11.2697009	1.801230293	7.9724E-60
TSC22D3	8.15507655	8.15582559	8.08787499	10.3496632	10.0328546	10.1057558	2.121153269	7.4913E-59
SRXN1	11.0397501	11.0734887	11.0617185	11.9822893	11.9529204	11.9672371	0.915717638	2.2186E-58
HSPB8	9.50145414	9.37301832	9.4728103	11.0043908	10.8363725	10.9977332	1.526942659	4.0135E-57
CDCA8	11.7095192	11.6628488	11.6704069	10.6987828	10.6596199	10.6258375	-1.029805887	4.8621E-56
TCN1	5.7318604	5.69890554	5.86403913	8.41872666	8.34145689	8.33059093	3.143749278	3.3044E-55
HNRNPA1	13.7947354	13.8062144	13.7488878	12.8080815	12.6911108	12.7661888	-1.030256582	1.8923E-54
MTHFD1	12.6237532	12.5768163	12.5280876	11.1561491	11.3730666	11.2844319	-1.309232954	2.5551E-48
TYMS	12.3820558	12.3720416	12.4299581	11.3290052	11.4149583	11.4254927	-1.009667497	7.3739E-48
MMP3	11.0667401	11.0552733	10.9913557	12.3111514	12.1812665	12.2348502	1.213428303	1.8969E-47
RHOB	8.73453197	8.82144644	9.03863955	10.6008592	10.6449222	10.7383232	1.840042909	1.4736E-45
COL4A2	13.3394968	13.3636793	13.3479521	12.222833	12.384201	12.2458451	-1.067097697	1.2954E-44
CDH4	12.0209291	12.0188133	12.027984	10.8570193	10.7821834	10.6853283	-1.257154525	2.1479E-44
LOC105370526	6.12223629	5.85379722	6.11763792	8.17306789	8.13663593	8.12375092	2.521768204	2.1479E-44
NPM1	15.5573695	15.6004562	15.526886	14.662501	14.6703444	14.7359382	-0.872440092	4.7317E-44
GABARAPL1	9.46356366	9.44970328	9.34449657	10.5328066	10.5476746	10.628559	1.176253598	1.0711E-43

Supplementary Table 6: Pearson correlation between *MIR22HG* and *cMYC* target expression

	METABRIC (TNBC)		TCGA (TNBC)	
cMYC target	Correlation vs MIR22HG	p-value	Correlation vs MIR22HG	p-value
BUB3	-0.209	0.000175	-0.199	0.00794
CAD	-0.32	5.28E-09	-0.219	0.00333
CBX3	-0.304	3.11E-08	-0.422	5.07E-09
CCT2	-0.18	0.000124	-0.347	2.26E-06
CCT5	-0.166	0.00298	-0.35	1.80E-06
CDC20	-0.327	2.16E-09	-0.364	6.25E-07
CDC45	-0.419	5.08E-15	-0.475	2.31E-11
EIF4H	-0.191	0.000598	-0.059	0.435
GNL3	-0.169	0.00244	-0.14	0.0623
HDAC2	-0.209	0.000175	-0.448	3.89E-10
HNRNPA1	-0.201	0.000309	-0.446	5.13E-10
HNRNPA2B1	-0.171	0.00219	-0.454	2.19E-10
HNRNPA3	-0.298	5.81E-08	-0.406	2.08E-08
HNRNPC	-0.0275	0.625	-0.453	2.32E-10
HNRNPD	-0.231	3.20E-05	-0.453	2.37E-10
HPRT1	-0.138	0.0134	-0.368	4.68E-07
HSP90AB1	-0.394	2.80E-13	-0.193	0.0102
HSPD1	-0.33	1.50E-09	-0.352	1.54E-06
KPNA2	-0.104	0.0647	-0.374	2.88E-07
LDHA	-0.0549	0.328	-0.0631	0.404
MCM2	-0.483	4.40E-20	-0.514	2.61E-13
MCM6	-0.376	3.63E-12	-0.499	1.52E-12
NAP1L1	0.0261	0.642	-0.316	1.78E-05
NPM1	-0.157	0.00488	-0.366	5.55E-07
ODC1	-0.243	1.12E-05	-0.399	3.92E-08
PCNA	-0.181	0.00115	-0.493	3.10E-12
PGK1	0.0583	0.299	0.0584	0.44
PSMD1	-0.087	0.121	-0.171	0.0227
PSMD14	-0.0766	0.172	-0.252	0.000705
RRM1	-0.403	6.75E-14	-0.486	7.31E-12
SERBP1	-0.298	6.04E-08	-0.499	1.56E-12
SET	-0.219	7.91E-05	-0.475	2.42E-11
SF3B3	-0.318	6.31E-09	-0.393	6.38E-08
SRSF1	-0.248	7.65E-06	-0.442	7.67E-10
SRSF2	-0.209	0.000174	-0.298	5.56E-05
SRSF3	-0.0566	0.314	-0.391	7.71E-08
SRSF7	-0.146	0.00898	-0.406	2.03E-08
TARDBP	-0.122	0.0293	-0.476	2.22E-11
TYMS	-0.264	1.74E-06	-0.519	1.40E-13
TCP1	-0.0552	0.325	-0.223	0.00286
VDAC1	-0.147	0.00848	-0.129	0.0881
