# Supplementary material for: Prolyl hydroxylase substrate adenylosuccinate lyase is an oncogenic driver in triple negative breast cancer
Source: Nat Commun. 2019 Nov 15;10:5177. doi: 10.1038/s41467-019-13168-4 (PMC6858455; doi:10.1038/s41467-019-13168-4)
Supplement: Supplementary file 3 — Reporting Summary [file 41467_2019_13168_MOESM3_ESM.pdf]

nature research

Corresponding author(s): DBPR NCOMMS-19-04181-T

Last updated by author(s): Oct 14, 2019

Reporting Summary

Nature Research wishes to improve the reproducibility of the work that we publish. This form provides structure for consistency and transparency in reporting. For further information on Nature Research policies, see Authors & Referees and the Editorial Policy Checklist.

Statistics

For all statistical analyses, confirm that the following items are present in the figure legend, table legend, main text, or Methods section.

n/a	Confirmed	
☐	☒	The exact sample size (*n*) for each experimental group/condition, given as a discrete number and unit of measurement
☐	☒	A statement on whether measurements were taken from distinct samples or whether the same sample was measured repeatedly
☐	☒	The statistical test(s) used AND whether they are one- or two-sided<br>*Only common tests should be described solely by name; describe more complex techniques in the Methods section.*
☒	☐	A description of all covariates tested
☒	☐	A description of any assumptions or corrections, such as tests of normality and adjustment for multiple comparisons
☐	☒	A full description of the statistical parameters including central tendency (e.g. means) or other basic estimates (e.g. regression coefficient) AND variation (e.g. standard deviation) or associated estimates of uncertainty (e.g. confidence intervals)
☐	☒	For null hypothesis testing, the test statistic (e.g. *F*, *t*, *r*) with confidence intervals, effect sizes, degrees of freedom and *P* value noted<br>*Give P values as exact values whenever suitable.*
☒	☐	For Bayesian analysis, information on the choice of priors and Markov chain Monte Carlo settings
☒	☐	For hierarchical and complex designs, identification of the appropriate level for tests and full reporting of outcomes
☒	☐	Estimates of effect sizes (e.g. Cohen's *d*, Pearson's *r*), indicating how they were calculated

Our web collection on statistics for biologists contains articles on many of the points above.

Software and code

Policy information about availability of computer code

Data collection	DESeq2, GSEA
Data analysis	Prism 5 (Graphpad); Excel (Microsoft)

For manuscripts utilizing custom algorithms or software that are central to the research but not yet described in published literature, software must be made available to editors/reviewers. We strongly encourage code deposition in a community repository (e.g. GitHub). See the Nature Research guidelines for submitting code & software for further information.

Data

Policy information about availability of data

All manuscripts must include a data availability statement. This statement should provide the following information, where applicable:

- Accession codes, unique identifiers, or web links for publicly available datasets
- A list of figures that have associated raw data
- A description of any restrictions on data availability

The RNA-seq dataset generated for this study has been deposited to the GEO with Study Accession number GSE136414 [https://www.ncbi.nlm.nih.gov/geo/query/acc.cgi?acc=GSE136414]. The mass spectrometry proteomics data for substrate-trapping interaction analysis have been deposited to the ProteomeXchange Consortium via the PRIDE partner repository with the dataset identifiers PXD015787 and PXD015790. The mass spectrometry proteomics data for ADSL hydroxyproline analysis have been deposited to the ProteomeXchange Consortium via the PRIDE partner repository with the dataset identifier PXD015773. All other relevant data supporting the main findings of the present study are available throughout the article, its Supplementary Information files and the source data file or from the corresponding author upon reasonable request. The source data underlying Figures 1 d-i, 2 b-h, 3 b, c, e, f, 4a, c-e, g, 5 d-g, i, 6 b, c, e-i, k-n, Supplementary Figures 1 d, e, f, 2 b-j, 3 b-d, 4 a-e, 5 a, b, d, f-h, j-l and 6 a, c-e are provided as a Source Data File.

Field-specific reporting

Please select the one below that is the best fit for your research. If you are not sure, read the appropriate sections before making your selection.

☒ Life sciences ☐ Behavioural & social sciences ☐ Ecological, evolutionary & environmental sciences

For a reference copy of the document with all sections, see nature.com/documents/nr-reporting-summary-flat.pdf

Life sciences study design

All studies must disclose on these points even when the disclosure is negative.

Sample size	No statistical methods were used to predetermine sample size. The number of recordings for each experiment was based on the experimenter's experience and data variability. The major findings were tested on multiple cell lines and obtained with orthogonal approaches in order to increase the robustness of the data.
Data exclusions	Experiments where the positive and negative controls did not work were excluded.
Replication	All experiments were successfully reproduced with similar results.
Randomization	The experiments were not randomized.
Blinding	Data collection of animal studies (described in fig. 3 and supplementary fig. 3), genomics (Figs. 2a, 2b, 6d, supplementary 2a, supplementary 6b, supplementary tables 3 and 6), metabolomics (fig. 5a, supplementary figs 4e and 5a-c) and nucleotide amount (supplementary fig. 5h) was performed by blinded experimenters. The rest of the experiments were performed by not blinded experimenters.

Reporting for specific materials, systems and methods

We require information from authors about some types of materials, experimental systems and methods used in many studies. Here, indicate whether each material, system or method listed is relevant to your study. If you are not sure if a list item applies to your research, read the appropriate section before selecting a response.

Materials & experimental systems

n/a	Involved in the study	
☐	☒	Antibodies
☐	☒	Eukaryotic cell lines
☒	☐	Palaeontology
☐	☒	Animals and other organisms
☒	☐	Human research participants
☒	☐	Clinical data

Methods

n/a	Involved in the study	
☒	☐	ChIP-seq
☒	☐	Flow cytometry
☒	☐	MRI-based neuroimaging

Antibodies

Antibodies used	Rabbit antibody against ADSL (HPA000525) and mouse antibodies against vinculin (V9131) and alpha-tubulin (T9026) were from Sigma-Aldrich. Rabbit anti-hydroxyproline (ab37067) and APRT (ab91428) antibodies were from Abcam. Rabbit antibodies against HIF-1α (14179), cMYC (5605), RRM1 (8637), 4EBP1 (9644), AMPK (5832), P-AMPK (2535), P-4EBP1 (2855), FLAG (14793), HA (3724) and GST (2625) tags were from Cell Signaling Technology. Mouse antibody against HA tag (901501) was from BioLegend. Mouse antibody against vimentin (550513) was from BD Biosciences. Rabbit antibody against EglN2 (NB100-310) was from Novus Biologicals. Mouse antibodies against RRM2 (sc-376973), CAD (sc-376072), TYMS (sc-390945), DHODH (sc-166348), GMPS (sc-376163) and β actin (sc-47778) were from Santa Cruz Biotechnology. Rabbit antibody against CTPS1 (A304-543A), peroxidase conjugated goat anti-mouse (31430) and anti-rabbit (31460) secondary antibodies were purchased from Thermo Fisher Scientific.
Validation	Rabbit antibody against ADSL (HPA000525) was validated by using ADSL knockout, as well as ADSL overexpressing cell lines (see figs. 2C and 2F as an example). Rabbit anti-hydroxyproline (ab37067) antibody was validated by overexpressing the prolyl hydroxylase EglN2, and by inhibiting it with the hydroxylase inhibitor DMOG (see figs. 4C and 4D as an example). Rabbit anti-APRT (ab91428) antibody was validated by using shRNA against APRT (see supplementary fig. S5L). Rabbit antibody against HIF-1α (14179) was validated by using hypoxia (1% O_2) (see fig. 1A and 1B as an example). Rabbit antibody against EglN2 (NB100-310) was validated using sgRNAs (see supplementary figs. S1D and S6E as example). Mouse antibody against RRM2 (sc-376973) and rabbit antibody against cMYC (5605) were validated by using overexpression vectors (results not shown in the paper; can be provided upon request).

Eukaryotic cell lines

Policy information about cell lines

Cell line source(s)	MDA-MB-231, MDA-MB-436, MCF7, T47D, MDA-MB-468, HMLE and MCF10A were from ATCC, 293T were from UNC Tissue Culture Facility (TCF)
Authentication	Cell lines not obtained from ATCC (i.e. 293T) were authenticated by UNC Tissue culture facility (TCF)
Mycoplasma contamination	Cell lines were tested negative by using the mycoplasm detection kit.
Commonly misidentified lines (See ICLAC register)	N/A

Animals and other organisms

Policy information about studies involving animals; ARRIVE guidelines recommended for reporting animal research

Laboratory animals	Six-week old female NOD SCID Gamma mice (NSG, Jackson lab)
Wild animals	*Provide details on animals observed in or captured in the field; report species, sex and age where possible. Describe how animals were caught and transported and what happened to captive animals after the study (if killed, explain why and describe method; if released, say where and when) OR state that the study did not involve wild animals.*
Field-collected samples	*For laboratory work with field-collected samples, describe all relevant parameters such as housing, maintenance, temperature, photoperiod and end-of-experiment protocol OR state that the study did not involve samples collected from the field.*
Ethics oversight	All animal experiments were in compliance with National Institutes of Health guidelines and were approved by the University of North Carolina at Chapel Hill Animal Care and Use Committee.

Note that full information on the approval of the study protocol must also be provided in the manuscript.
